# Metastasis risk stratification and response prediction through dynamic viable circulating tumor cell counts for rectal cancer in a neoadjuvant setting

**DOI:** 10.1002/cam4.5860

**Published:** 2023-04-04

**Authors:** Wen‐Yang Liu, Wen Zhang, Yuan Tang, Si‐Lin Chen, Ning Li, Jun‐Qin Lei, Jin‐Ming Shi, Shu‐Lian Wang, Ye‐Xiong Li, Kai‐Tai Zhang, Jing Jin

**Affiliations:** ^1^ Department of Radiation Oncology National Cancer Center/National Clinical Research Center for Cancer/Cancer Hospital, Chinese Academy of Medical Sciences and Peking Union Medical College Beijing China; ^2^ Department of Immunology National Cancer Center/National Clinical Research Center for Cancer/Cancer Hospital, Chinese Academy of Medical Sciences and Peking Union Medical College Beijing China; ^3^ Department of Radiation Oncology National Cancer Center/Cancer Hospital & Shenzhen Hospital, Chinese Academy of Medical Sciences and Peking Union Medical College Shenzhen China

**Keywords:** circulating tumor cells, locally advanced rectal cancer, metastasis, neoadjuvant treatment, preoperative chemoradiation

## Abstract

**Purpose:**

Distant metastasis (DM) and neoadjuvant treatment response prediction remain critical challenges in the management of locally advanced rectal cancer (LARC). The aim of this study was to investigate the clinical relevance of viable circulating tumor cells (CTCs) for DM or response in patients with LARC in a neoadjuvant setting.

**Methods:**

The detection of viable CTCs at different stages of treatment was planned for consecutive patients from a prospective trial. The Kaplan–Meier method, Cox proportional hazards model, and logistic regression model were utilized to analyze factors associated with DM or pathological complete response (pCR) and clinical complete response (cCR).

**Results:**

Between December 2016 and July 2018, peripheral blood samples from 83 patients were collected before any treatment (median follow‐up time, 49.3 months). CTCs were present in 76 of 83 patients (91.6%) at baseline, and more than three CTCs detected in the blood sample was considered high risk. Only the CTC risk group was significantly associated with 3‐year metastasis‐free survival (MFS) (high risk vs. low risk, 57.1% (95% CI, 41.6–72.6) vs. 78.3% (95% CI, 65.8–90.8), *p* = 0.018, log‐rank test). When all the important variables were entered into the Cox model, the CTC risk group remained the only significant independent factor for DM (hazard ratio (HR), 2.74; 95% CI, 1.17–6.45, *p* = 0.021). The pCR and continuous cCR rates were higher in patients with a decreased number of CTCs of more than one after radiotherapy (HR, 4.00; 95% CI, 1.09–14.71, P = 0.037).

**Conclusions:**

The dynamic detection of viable CTCs may strengthen pretreatment risk assessment and postradiotherapy decision making for LARC. This observation requires further validation in a prospective study.

## 1 INTRODUCTION

Colorectal cancer (CRC) is one of the most common malignancies and causes of cancer‐related deaths worldwide.^1^ Although locoregional control is satisfactory for locally advanced rectal cancer (LARC) in a neoadjuvant setting with total mesorectal excision (TME), the survival rate has not improved because distant metastasis (DM) (17%–28%) has not been definitively resolved to date.^2^, ^3^, ^4^

Although chemotherapy can theoretically eliminate subclinical micrometastases, several studies and meta‐analyses have shown that adjuvant chemotherapy with fluorouracil alone does not improve survival in rectal cancer patients after neoadjuvant chemoradiation (CRT),^5^, ^6^, ^7^ even with the addition of oxaliplatin.^8^ Induction chemotherapy is currently being studied due to its better compliance and timing advantage, but its efficacy for DM remains disappointing.^9^ Recently, Bahadore et al.^3^ reported that the cumulative probability of DM was decreased to 20.0% from 26.8% for patients with unfavorable clinical features through short‐course radiotherapy with total neoadjuvant chemotherapy. Moreover, the UNICANCER‐PRODIGE 23 study demonstrated that intensified neoadjuvant chemotherapy using FOLFIRINOX significantly improved disease‐free survival^4^; however, there was a lack of an overall survival (OS) benefit in both of these trials. The evidence above shows that it is difficult to use initial clinical or pathological factors to determine the risk of DM in patients, and thus, these factors can hardly be used to accurately select candidates who truly need chemotherapy. Nevertheless, the number of robust biomarkers for screening patients at high risk of DM is also limited, and thus, a way to identify patients who need intensive treatment is urgently needed.

In recent years, increasing evidence has shown that liquid biopsy, which encompasses circulating tumor DNA^10^ and circulating tumor cells (CTCs),^11^ can be used as a prognostic tool in a variety of tumors, with the advantages of minimal invasiveness and easy dynamic monitoring. Extensive studies have been conducted in CRC, with a definite prognostic effect reported,^12^, ^13^ and long‐term efficacy can be predicted in both metastatic CRC (mCRC) and nonmetastatic CRC after surgery; however, only a few studies^14^, ^15^, ^16^, ^17^, ^18^, ^19^ have reported the impact of CTCs on the short‐term efficacy of preoperative CRT. Thus, the impact of baseline CTCs on long‐term oncological results for LARC has not yet been adequately characterized in a neoadjuvant setting.^16^

Immortalization is one of the basic characteristics of tumors, and it is realized through telomerase reverse transcriptase (TERT). The core of the approach we used in this study is the positive labeling of TERT‐positive cells. In our previous study, high sensitivity and accuracy for CTC detection were achieved with tumor‐selective replication herpes simplex virus‐based technology rather than CellSearch,^20^ and this method has shown clinical application value in early diagnosis and prognosis prediction.^20^, ^21^, ^22^, ^23^ Therefore, based on a subset of the phase III trial, our primary aim was to investigate whether viable CTCs detected by this in‐house method could be a prognostic factor for DM in patients with LARC in a neoadjuvant setting.

## 2 METHODS

### 2.1 Study design and enrollment

Patients from a single principal center in a multicenter, open‐label, prospective phase II/III randomized trial who agreed to attend a translational study session were included in this analysis. In brief, the patients were randomized to receive short‐course preoperative radiotherapy (SCPRT, 5 Gy × 5 alone) with neoadjuvant chemotherapy (NCT) (4 cycles of the XELOX regimen) (experimental group) or preoperative long‐course chemoradiotherapy (2 Gy × 25 with capecitabine) (control group) for middle‐lower LARC. The inclusion criteria, treatment, and follow‐up details were described in our previous report.^24^

**FIGURE 1:**
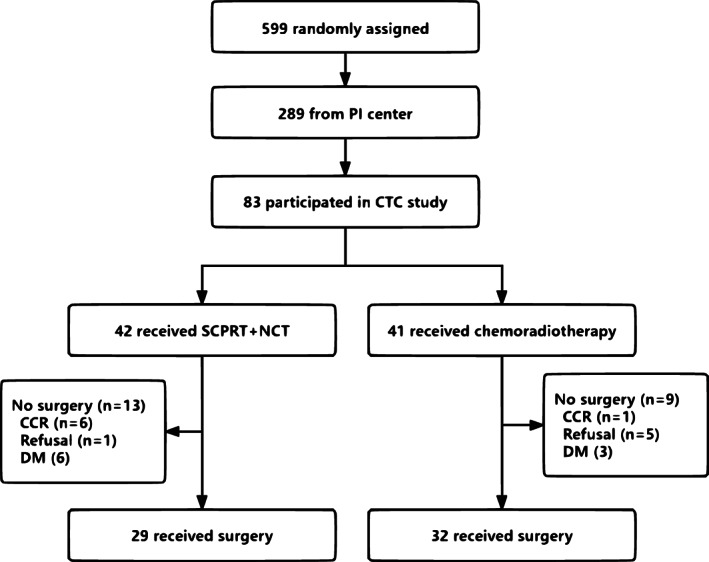
CONSORT diagram. The inclusion process of the patients for this translational study. PI, principal investigator; CTC, circulating tumor cell; SCPRT, short‐course preoperative radiotherapy; CCR, complete clinical response; DM, distant metastasis; n, number.

The exploratory study with respect to CTCs was prospectively designed with the hypothesis that if the hazard ratio (HR) for metastasis‐free survival (MFS) of the high‐risk patients defined by CTC counts was more than 2.5 compared to that of the low‐risk patients, a two‐sided log‐rank test with an overall sample size of 78 subjects (39 each in the high‐risk and low‐risk groups) would achieve 80.1% power at a 0.050 significance level. The detection of CTCs at different stages of treatment was also planned (before and after radiotherapy for all patients and during and after NCT for the SCPRT group). A total of 599 patients have been examined to date, and among the 289 patients treated at the principal center, 83 willing to participate in this translational part of the study were consecutively included between December 2016 and June 2018 (all of whom were willing to participate in this translational study and to provide blood samples, without any other selection bias) (Figure 1). Blood was drawn at baseline, at the end of chemoradiotherapy for the control group or 1 week after SCPRT for the experimental group. Data were collected and located in the electronic data capture system. This article was written according to the REMARK criteria^25^ for the reporting of tumor markers.

### 2.2 Viable CTC detection method

Peripheral blood samples (4 mL) were prepared in heparinized tubes and incubated with lysis buffer (NH_4_, 0.15 M; EDTA, 0.1 mM; KHCO_3_, 10 mM; pH = 7.2) at room temperature for 5 min. After centrifugation, the supernatant was discarded, and the cell pellets were washed 2× with PBS. The oHSV1‐hTERT‐GFP virus used in this study, in which the endogenous ICP4 promoter is replaced with the hTERT promoter, has been described in our previous work.^26^ Following centrifugation, cells were resuspended and transduced with oHSV1‐hTERT‐GFP at a multiplicity of infection of 1 at 37°C in a humidified atmosphere of 5% CO_2_ for another 24 h. Thereafter, the cells were collected, and 200 μL of PE‐Cy5 mouse anti‐human CD45 (HI30, BD) was added, followed by incubation in the dark at room temperature for 30 min. After one wash with PBS, the cells were resuspended in 1 mL of PBS. The detection of CD45‐/GFP+ cells from blood samples was performed via flow cytometry (Merck Millipore or BD). CD45‐/GFP+ cells were recorded as positive results.

### 2.3 FlowSight confirmation of the CTCs detected by oHSV1‐hTERT‐GFP

For flow imaging, samples were processed according to the procedure and incubated with the eFluor405‐CD45 antibody (clone: HI30, BioLegend) and APC‐EpCAM antibody (clone: CO17‐1A, BioLegend). Then, CTCs were detected by the ImageStreamX® Mark II system (Amnis). CD45‐/GFP+/EpCAM+ cells in blood samples were considered CTC‐positive cells.

### 2.4 Statistical analysis

IBM SPSS Statistics 17.0 software (SPSS Inc.) was used to manage the data. OS, MFS, and locoregional recurrence (LR) were defined as previously reported.^24^ Progression‐free survival (PFS) was defined as the time from randomization to a diagnosis of local recurrence or progression (the first occurrence of locoregional failure for patients who underwent radical surgery), DM, or death, whichever was observed first. Clinical characteristics were analyzed by descriptive statistics. We applied Fisher's exact test to evaluate the potential associations between CTC status and these parameters. Patient OS, PFS, and MFS were analyzed using the Kaplan–Meier method and tested by the log‐rank test. The Cox proportional hazards model was adopted for intergroup comparisons, and multivariate analysis was performed to assess the independent influence of CTCs and other covariates on tumor events, which are expressed in the form of the HR and 95% confidence interval (CI). A logistic regression model was utilized to analyze factors associated with the occurrence of pathological complete response (pCR) and clinical complete response (cCR). The Mann–Whitney test was used to compare the numerical distribution of different unpaired groups. The Youden index was used in conjunction with receiver operating characteristic (ROC) curve analysis to determine the optimum cutoff of numeric predictor parameters. Significance was considered with *p* < 0.05 in two‐tailed tests. All authors had access to the study data and reviewed and approved the final manuscript.

## 3 RESULTS

### 3.1 Baseline patient characteristics, CTC distribution and verification

Between December 2016 and July 2018, peripheral blood samples from 83 patients were collected before any treatment, with a median follow‐up time of 49.3 months as of December 2021 (range, 9.3–58.7 months). There were 55 male (66.3%) and 28 female (33.7%) patients, with a median age of 55 years (range, 29–69 years). The numbers of patients with tumors staged IIA, IIC, IIIB and IIIC were 13 (15.7%), 1 (1.2%), 56 (67.5%), and 13 (15.7%), respectively. In total, 57 (68.7%) and 43 (51.8%) patients had tumors with a positive mesorectal fascia status and a positive extramural vascular invasion (EMVI) status, respectively. CTCs were present at baseline in 76 of 83 patients (91.6%), ranging from 1 to 18 cells (median = 3 cells).

**FIGURE 2:**
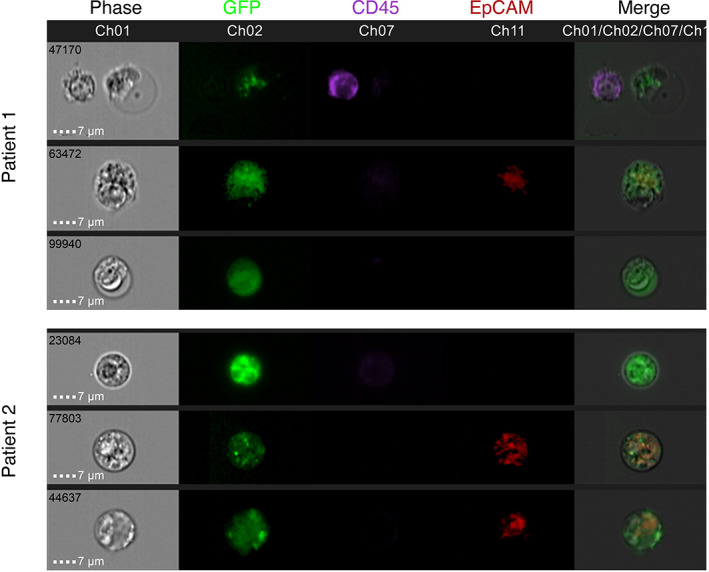
CTCs detected by the FlowSight system. Bright‐field, eFlour405‐CD45 (purple), hTERTp‐GFP (green), APC‐EpCAM (red), and merged digital images are shown for CTCs. CTCs derived from patients by GFP with or without the EpCAM marker. CTCs were defined as CD45‐/GFP+/EpCAM+ or CD45‐/GFP+/EpCAM‐. WBCs could only be marked with CD45 (the first column of patient 1, both white blood cells and tumor cells were collected). Scale bar, 7 μm. In patient 1 and patient 2, 4. and 5 CTCs were detected, respectively.

To verify the CTCs, FlowSight imaging was performed. As shown in Figure 2, CTCs with GFP expression under control of the hTERT promoter were observed with or without EpCAM expression (CD45‐/GFP+/EpCAM+ or CD45‐/GFP+/EpCAM‐). Additionally, white blood cells (WBCs) only expressed CD45 (CD45+/GFP‐/EpCAM‐). We found that there was heterogeneity in CTCs among different patients or in the same patient with or without EpCAM expression. CTCs are generally large in volume and can be labeled by GFP under the control of the hTERT promoter. Moreover, CTCs showed heterogeneity in EpCAM expression or nonexpression (CD45‐GFP + EpCAM+ or CD45‐GFP + EpCAM‐). Even in the same patient, there were heterogeneous CTCs.

### 3.2 Treatments

Eighty‐three patients were randomly assigned: 42 (51.8%) received SCPRT plus chemotherapy and 41 (48.2%) received conventional preoperative concurrent chemoradiotherapy. Finally, 61 patients underwent surgery; the reasons the 22 patients did not undergo surgery were cCR (*n* = 7), refusal (*n* = 6), and DM before surgery (n = 9) (for details, see Table S1). During the median follow‐up time of 49.3 months, 19 deaths (12 due to metastasis, five due to locoregional failure, and two due to comorbidities), eight local recurrences, two local progression cases, and 27 metastases (22 with liver or lung metastasis only, one with both, and four with metastasis in another location) were observed.

### 3.3 Univariate survival analysis

The 3‐year OS rates were 86.9% (95% CI, 78.5–95.3) for patients who underwent surgery and 68.2% (95% CI, 48.8–87.6) (*p* = 0.015) for patients who did not undergo surgery, and the 3‐year PFS rates were 63.4% (95% CI, 51.2–75.6) and 45.5% (95% CI, 24.7–66.3) (*p* = 0.071), respectively.

**FIGURE 3:**
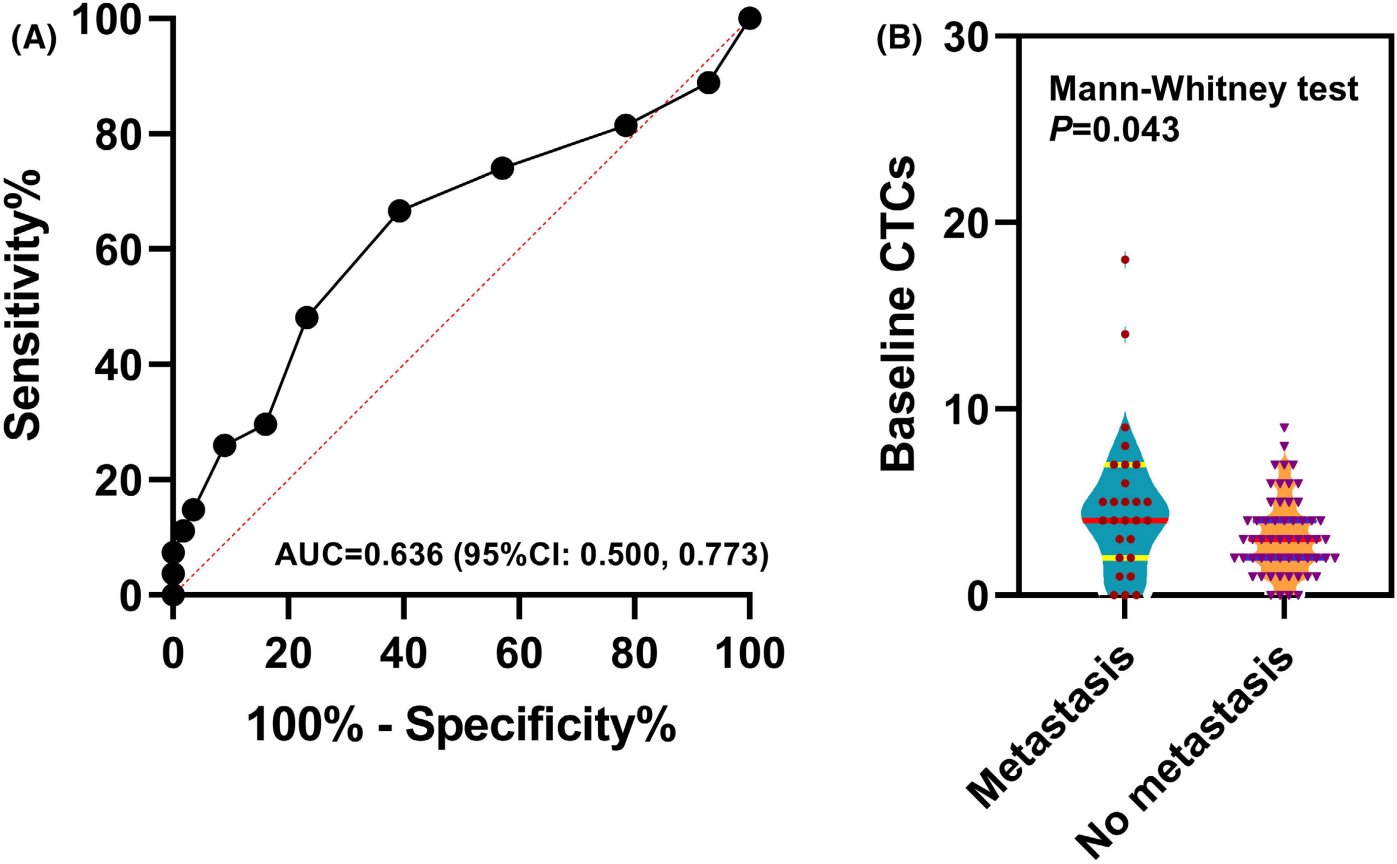
(A), The ROC curve based on baseline CTC count for metastasis prediction. (B), The baseline CTC count distribution between the groups of patients with metastasis and without metastasis; Mann–Whitney test for median comparison.

With a cut‐off count of three, CTCs at baseline could be used to identify the occurrence of DM, reaching the highest Youden index of 0.274, with a sensitivity of 66.7% (95% CI, 53.0%–80.4%) and a specificity of 60.7% (95% CI, 47.0%–74.4%) (Figure 3A). The significant difference of baseline CTC count distribution between the groups of patients with metastasis and without metastasis was observed (*p* = 0.043, Mann–Whitney test, Figure 3B). Therefore, the patients were divided into a high‐risk group (CTCs >3) and a low‐risk group (CTCs ≤3) according to the number of CTCs at baseline. The baseline characteristics and treatment distributions were well balanced between the two groups (Table 1).

**TABLE 1:** Baseline characteristics and neoadjuvant treatments administered to the patients according to the CTC risk group.

Variable	Low‐risk group CTCs≤3 (No. and (%)) *n* = 43	High‐risk group CTCs>3 (No. and (%)) *n* = 40	*p*
Age			
≥60	13 (30.2)	15 (37.5)	0.497
<60	30 (69.8)	25 (62.5)	
Sex			
Male	28 (65.1)	27 (67.5)	1.000
Female	15 (34.9)	13 (32.5)	
Tumor location			
Middle	13 (30.2)	8 (20.0)	0.321
Lower	30 (69.8)	32 (80.0)	
Tumor stage by MRI			
T3	41 (95.3)	39 (97.5)	1.000
T4	2 (4.7)	1 (2.5)	
N0	8 (18.6)	6 (15.0)	0.671
N1	13 (30.2)	16 (40.0)	
N2	22 (51.2)	18 (45.0)	
MRF status by MRI			
Negative	13 (30.2)	13 (32.5)	1.000
Positive	30 (69.8)	27 (67.5)	
EMVI status by MRI			
Negative	25 (58.1)	15 (37.5)	0.079
Positive	18 (41.9)	25 (62.5)	
Treatment modality			
SCPRT with nCT	24 (55.8)	18 (45.0)	0.383
CRT	19 (44.2)	22 (55.0)	

*Note*: The patients were divided into a high‐risk group (CTCs >3) and a low‐risk group (CTCs ≤3) according to CTCs at baseline.

Abbreviations: CTC, circulating tumor cell; EMVI, extramural vascular invasion; MRF, mesorectal fascia; nCT, neoadjuvant chemotherapy; *N*, number; SCPRT, short‐course preoperative radiotherapy.

**FIGURE 4:**
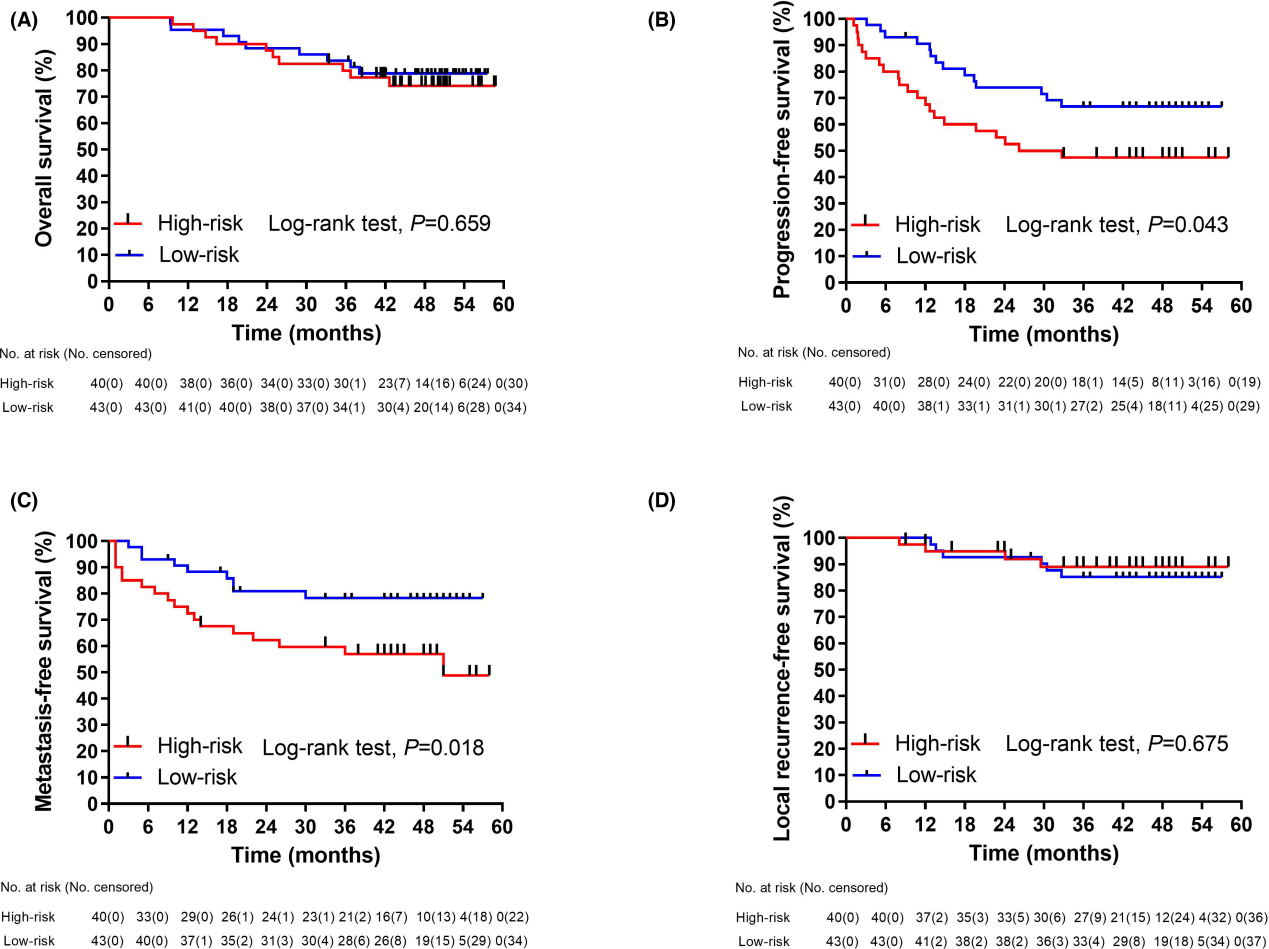
Survival curves by the Kaplan–Meier method according to the CTC risk group. (A), overall survival; (B), progression‐free survival; (C), metastasis‐free survival; (D), local recurrence‐free survival.

Ten and nine deaths were observed in the high‐risk and low‐risk groups, respectively, while four and six patients presented with LR or progression, respectively. No significant differences in OS (*p* = 0.659, Figure 4A) or local recurrence‐free survival (LRFS; *p* = 0.675, Figure 4D) were found between the two groups.

PFS was significantly worse in the high‐risk group than low‐risk group (Figure 4B). With respect to DM, 18 and nine patients in the high‐risk and low‐risk groups, respectively, experienced events (3‐year MFS of 57.1% [95% CI, 41.6–72.6] vs. 78.3% [95% CI, 65.8–90.8], *p* = 0.018, log‐rank test). Upon analyzing various relevant clinical factors (detailed MFS by each variable, see Table 2), MFS was only significantly associated with the CTC risk group (Figure 4C).

**TABLE 2:** Results from the univariate and multivariate Cox regression analyses for MFS.

Group	Univariate analysis		Multivariate analysis	
	HR (95% CI)	*p*	HR (95% CI)	*p*
Age: ≥60 vs. <60	0.64 (0.27–1.51)	0.306	0.56 (0.21–1.50)	0.247
Sex: Female vs. male	1.17 (0.54–2.56)	0.693	0.96 (0.39–2.37)	0.934
Tumor location: Lower vs. middle	0.79 (0.35–1.80)	0.573	0.68 (0.27–1.73)	0.421
MRF: Positive vs. negative	0.77 (0.35–1.69)	0.519	0.73 (0.30–1.77)	0.481
EMVI: Positive vs. negative	1.59 (0.74–3.43)	0.240	1.18 (0.48–2.87)	0.718
mrN: N2 vs. N0‐1	1.09 (0.51–2.32)	0.832	0.84 (0.34–2.04)	0.698
Neoadjuvant treatment: CRT vs. SCPRT+nCT	1.14 (0.54–2.43)	0.734	1.15 (0.51–2.60)	0.745
CTC risk: High vs. low	2.53 (1.14–5.65)	0.023	2.74 (1.17–6.45)	0.021

*Note*: The patients were divided into a high‐risk group (CTCs >3) and a low‐risk group (CTCs ≤3) according to CTCs at baseline. For Cox regression, the latter subgroup is the reference.

Abbreviations: CTC, circulating tumor cell; EMVI, extramural vascular invasion; HR, hazard ratio; MFS, metastasis‐free survival; MRF, mesorectal fascia; mrN, MRI nodal staging; *N*, number; nCT, neoadjuvant chemotherapy; SCPRT, short‐course preoperative radiotherapy.

### 3.4 Multivariate survival analysis

Although no other relevant factors were found through the univariate analysis, we incorporated some previously reported significant factors that may affect DM into the multivariate Cox model analysis. When the age group (above 60 years vs. below 60 years), sex, tumor location (middle vs. lower segment), MRI lymph node stage (N2 vs. N0‐1), mesorectal fascia (MRF), EMVI status, neoadjuvant treatment modality, and CTC risk group were included, the CTC risk group remained the only independent prognostic factor for DM, with a nearly threefold higher risk of DM in the high‐risk group than in the low‐risk group (HR, 2.74; 95% CI, 1.17–6.45, *p* = 0.021).

### 3.5 Relationship among postradiotherapy changes in CTCs, treatment response and prognosis

A total of 74 patients underwent a CTC examination after radiotherapy, with CTCs detected in 73 patients (98.8%) (median count = 2, range 0–9).

#### 3.5.1 Association between changes in the CTC count and neoadjuvant treatment response

**FIGURE 5:**
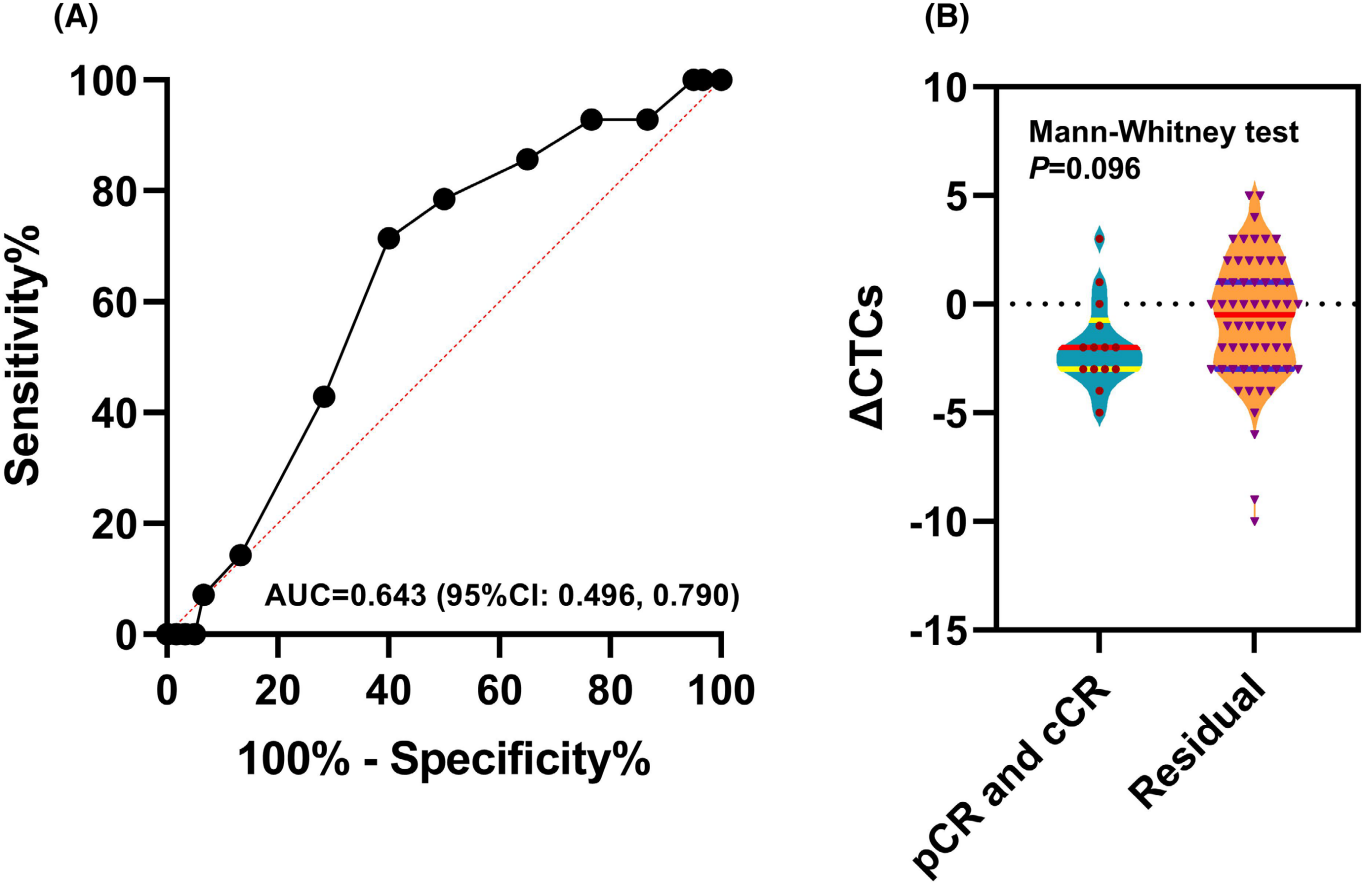
(A), The ROC curve based on ΔCTCs (the postradiotherapy change value of the CTC count from baseline) for pCR and cCR prediction (pCR, pathological complete response; cCR, complete clinical response). (B), The ΔCTC distribution between the groups of patients with and without pCR and cCR (residual); Mann–Whitney test for median comparison.

With a cut‐off count of −1, the reduction in CTCs at postradiotherapy versus baseline could be used to predict the occurrence of pCR and cCR, reaching the highest Youden index of 0.314, with a sensitivity of 60% (95% CI, 45.3%–74.7%) and a specificity of 71.4% (95% CI, 56.7%–84.1%; Figure 5A). No significant difference was observed in the ΔCTC distribution between the groups of patients with and without pCR and cCR (residual) (*p* = 0.096, Mann–Whitney test, Figure 3B). In total, 34 patients had a decreased number of CTCs of more than one (ΔCTCs <−1) (45.9%, Group A), and the other 40 patients had a decreased or even increased number of CTCs (54.1%, Group B). No association was observed between the change pattern of CTCs and the neoadjuvant modality. There were more pCR and continuous cCR cases in Group A (29.4% vs 10.0%, *p* = 0.041), while the common important clinical factors were balanced between these two groups, and no significant difference was found in survival outcomes (Table 3). In multivariate logistic regression analyses including important factors (*p* < 0.1), ΔCTCs <−1 remained significantly associated with pCR and cCR (HR = 4.00, 95% CI, 1.09–14.71, *p* = 0.037; Table 4).

**TABLE 3:** Clinical factors, response, and survival by postradiotherapy changes in CTCs.

Parameters	ΔCTCs<−1 (No. and %) (*n* = 34)	ΔCTCs≥ − 1 (No. and %) (*n* = 40)	*p*
Age			
≥60	10 (29.4)	13 (32.5)	0.775
<60	24 (70.6)	27 (67.5)	
Sex			
Male	23 (67.6)	25 (62.5)	0.644
Female	11 (32.4)	15 (37.5)	
Tumor location			
Middle	5 (14.7)	12 (30.0)	0.119
Lower	29 (85.3)	28 (70.0)	
Tumor stage by MRI			
T3	34 (100.0)	38 (95.0)	0.496
T4	0 (0.0)	2 (5.0)	
N0	5 (14.7)	6 (15.0)	0.999
N1	12 (35.3)	14 (35.0)	
N2	17 (50.0)	20 (50.0)	
MRF status by MRI			
Negative	9 (26.5)	16 (40.0)	0.220
Positive	25 (73.5)	24 (60.0)	
EMVI status by MRI			
Negative	14 (41.2)	22 (55.0)	0.236
Positive	20 (58.8)	18 (45.0)	
Treatment			
SCPRT+nCT	15 (44.1)	20 (50.0)	0.647
CRT	19 (55.9)	20 (50.0)	
Surgery	24 (70.6)	32 (80.0)	
Response			
cCR	5 (14.7)	1 (2.5)	0.088
pCR	5 (14.7)	3 (7.5)	0.458
pCR and cCR	10 (29.4)	4 (10.0)	0.041
3‐year survival (% and 95% CI)			
OS	82.2 (69.3–95.1)	82.5 (70.7–94.3)	0.672
PFS	61.8 (45.5–78.1)	59.1 (43.6–74.6)	0.990
LRFS	97.1 (91.4–100.0)	81.3 (68.8–93.8)	0.055
MFS	67.2 (51.3–83.1)	71.4 (57.1–85.7)	0.453

Abbreviations: cCR, complete clinical response; CRT, chemoradiation; CTC, circulating tumor cell; LRFS, local recurrence‐free survival; MFS, metastasis‐free survival.; nCT, neoadjuvant chemotherapy; No., number; OS, overall survival; pCR, pathological complete response; PFS, progression‐free survival; SCPRT, short‐course preoperative radiotherapy; ΔCTCs, the postradiotherapy change in the circulating tumor cell count from baseline.

**TABLE 4:** Results from the univariate and multivariate logistic regression analyses for pCR and cCR.

Group	Univariate analysis		Multivariate analysis	
	HR (95% CI)	*p*	HR (95% CI)	*p*
Age: ≥60 vs. <60	1.96 (0.63–6.11)	0.247		
Sex: Female vs. male	0.67 (0.19–2.32)	0.524		
Tumor location: Lower vs. middle	2.52 (0.52–12.23)	0.252		
MRF: Positive vs. negative	0.89 (0.27–2.94)	0.853		
EMVI: Positive vs. negative	0.78 (0.25–2.39)	0.660		
mrN: N2 vs. N0‐1	0.32 (0.09–1.12)	0.074	0.30 (0.08–1.13)	0.075
Neoadjuvant treatment: CRT vs. SCPRT+nCT	0.63 (0.20–1.96)	0.424		
ΔCTCs: <−1 vs. ≥ − 1	3.75 (1.05–13.34)	0.041	4.00 (1.09–14.71)	0.037

*Note*: For Cox regression, the latter subgroup is the reference.

Abbreviations: CTC, circulating tumor cell; EMVI, extramural vascular invasion; HR, hazard ratio; MFS, metastasis‐free survival; MRF, mesorectal fascia; mrN, MRI nodal staging; N, number; nCT, neoadjuvant chemotherapy; SCPRT, short‐course preoperative radiotherapy; ΔCTCs, the postradiotherapy change value of the circulating tumor cell count from baseline.

#### 3.5.2 Association between changes in the CTC count and survival

**FIGURE 6:**
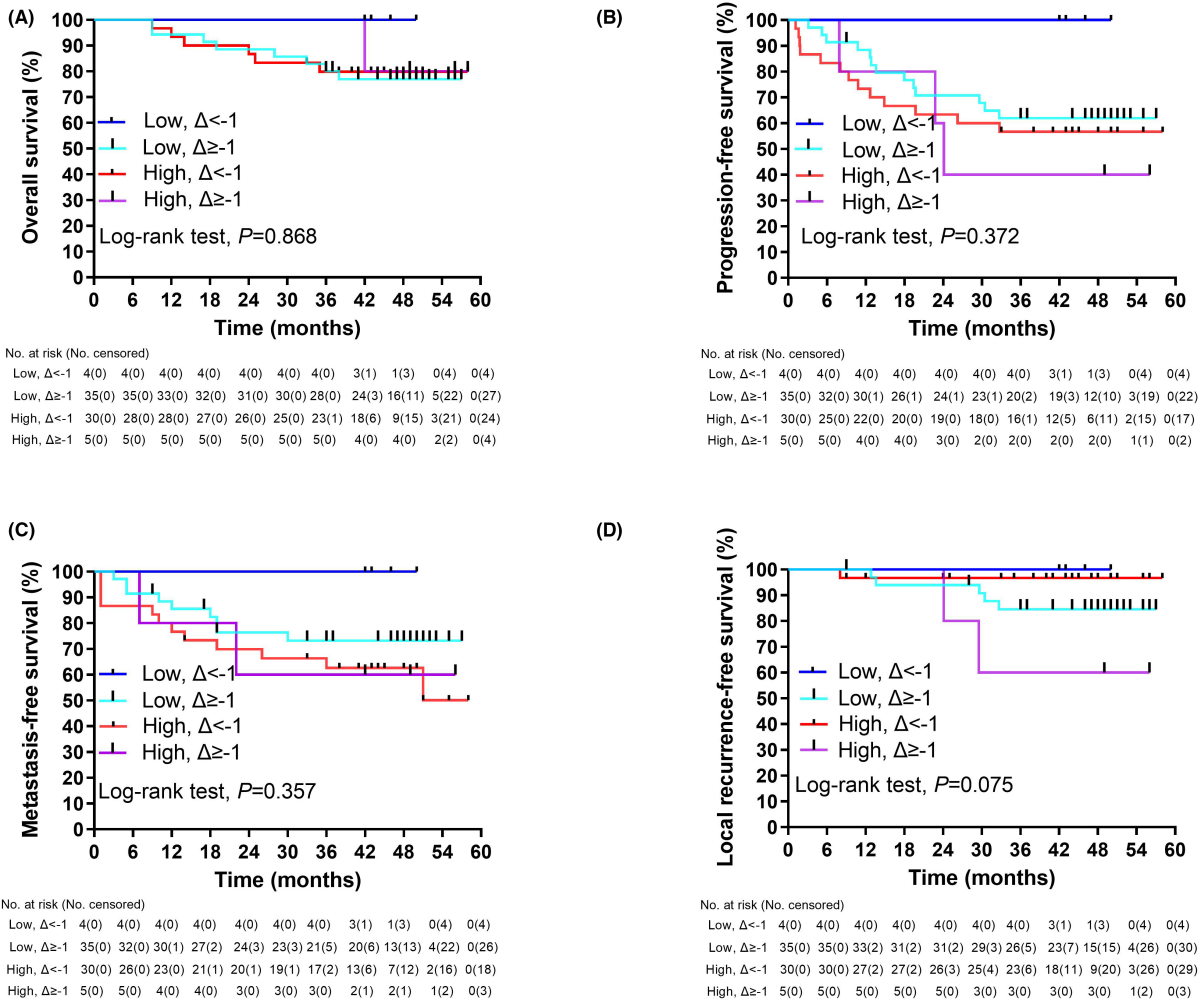
Survival curves by the Kaplan–Meier method according to the CTC risk group and the ΔCTCs (the postradiotherapy change value of the CTC count from baseline). Low, CTCs low‐risk; High, CTCs high‐risk; Δ < −1, a decreased number of CTCs more than 1 after radiotherapy; Δ ≥ −1, a decreased number of CTCs equal to or less than one after radiotherapy. (A), overall survival; (B), progression‐free survival; (C), metastasis‐free survival; (D), local recurrence‐free survival.

Between Groups A and B, the 3‐year survival outcomes were similar except for a better trend in LRFS (*p* = 0.055) (Table 3). Among the 39 low‐risk patients, four presented with ΔCTCs <−1, and none of the patients had any oncological event. Among the 35 high‐risk patients, 30 had ΔCTCs <−1. No significant difference was observed in the multiple group comparisons comparison for OS (Figure 6A), PFS (Figure 6B), MFS (Figure 6C), and LRFS (Figure 6D), or in the survival between these two subsets except for LRFS (*p* = 0.011, log‐rank test, Table 5).

**TABLE 5:** Survival by postradiotherapy changes in CTCs in the low‐risk and high‐risk groups.

3‐year survival	Low‐risk group, CTCs≤3 (% and 95% CI) *n* = 39		*p*	High‐risk group, CTCs>3 (% and 95% CI) n = 35		*p*
	ΔCTCs<−1 (*n* = 4)	ΔCTCs≥ − 1 (*n* = 35)		ΔCTCs<−1 (*n* = 30)	ΔCTCs≥ − 1 (*n* = 5)	
OS	100.0	80.0 (66.7–93.3)	0.309	79.9 (65.4–94.4)	80.0 (44.9–100.0)	0.879
PFS	100.0	61.9 (45.6–78.2)	0.170	56.7 (39.1–74.3)	40.0 (0–82.9)	0.640
LRFS	100.0	84.5 (72.0–97.0)	0.416	96.7 (90.2–100)	60.0 (17.1–100.0)	0.011
MFS	100.0	73.2 (58.1–88.3)	0.267	62.7 (45.1–80.3)	60.0 (17.1–100.0)	0.962

*Note*: The patients were divided into a high‐risk group (CTCs >3) and a low‐risk group (CTCs ≤3) according to CTCs at baseline. CTC, circulating tumor cell; ΔCTCs, the postradiotherapy change in the circulating tumor cell count from baseline.

Abbreviations: CI, confidence interval; LRFS, local recurrence‐free survival; MFS, metastasis‐free survival.; *n*, number; OS, overall survival; PFS, progression‐free survival.

## 4 DISCUSSION

With the application of TME and preoperative CRT, the local control of LARC is relatively satisfactory, while DM remains the main problem at present.^2^ For years, studies have failed to show significant efficacy for chemotherapy in preventing DM in LARC patients after preoperative CRT.^5^, ^6^, ^8^, ^9^ Only recently has a modest DM benefit been demonstrated through the randomized trials by Bahadore et al.^3^ and Conroy et al.^4^; meanwhile, the inability to accurately stratify patients at high risk for metastasis may be one of the main reasons limiting the efficiency of chemotherapy. According to an exploratory analysis of a subset of patients from phase III trials, the current study indicated that CTCs at baseline may help select candidates at high risk for DM in a neoadjuvant setting independent of other relevant clinical parameters.

Several previous studies showed an association between the change in CTC count and the treatment response in LARC patients treated with neoadjuvant therapy,^14^, ^15^ and this study also showed that more patients with ΔCTCs <−1 achieved cCR and pCR. Since CTCs detected by our method are more likely to reflect the grade of dissemination for viable tumor cells in the circulatory system, it is reasonable that a moderate association was observed only between changes in the CTC count and the local therapeutic response. More molecular markers may help improve the accuracy, as reported by Silva et al.^16^ and Flores et al.^17^

However, the majority of previous studies failed to show or did not report a relationship with long‐term prognosis, which may be related to the sensitivity of the methods used. CTCs were detected in 90% of the patients in the current study, while the detection rate in other studies that used the size‐based method was relatively low (19%).^14^ The difference in principle between the size‐based method adopted in previous studies and the tumor‐selective replication herpes simplex virus‐based technology adopted in this study may contribute to differences in the findings. The higher sensitivity and specificity of the virus‐based method can better reflect both the number and viability of CTCs, which are closely correlated with the dissemination ability of tumor cells, to indicate DM. This was confirmed in our previous report^20^ as well as in other studies on different tumor categories.^27^, ^28^ On the other hand, the generalizability of the cut‐off value was limited by the particular method, and standardized kit development should be considered in future research.

The novel finding that baseline CTCs have a prognostic effect on DM in LARC patients receiving neoadjuvant therapy in the current study is consistent with the findings from one study on advanced CRC^29^ and another on pancreatic cancer.^30^ In advanced CRC, size‐based methods can reveal the prognostic value of CTCs, possibly due to the relatively high number of CTCs in these populations. Cohen et al.^31^ found that CTCs at baseline detected by the CellSearch method were an independent prognostic factor of OS and PFS in advanced CRC. Moreover, the prognosis of high‐risk patients was better than that of nontransformed patients if they were converted to low‐risk patients after treatment. Both baseline values and changes in the CTC count were important factors for efficacy.^29^, ^31^ In our study, patients in the high‐risk group with ΔCTCs ≥−1 had the worst local prognosis.

The optimal strategy that can be used to control DM in LARC is still not ideal, although a decline of 6.8% in the cumulative probability of DM could be achieved in LARC patients with unfavorable clinical features through short‐course radiotherapy with total neoadjuvant chemotherapy,^3^ and an increase of 7.0% in the 3year MFS rate has been obtained through intensification of chemotherapy before preoperative CRT in patients with cT3 or cT4 M0 rectal cancer.^4^ The unsatisfactory effectiveness of systemic treatment and the lack of efficient biomarkers for metastasis risk stratification may have contributed to the current situation. In our study, the cumulative metastasis rate was approximately 40% for the high‐risk group, apparently higher than the rates of 25%–26.8% in the control groups of the UNICANCER‐PRODIGE 23 and RAPIDO trials.^3^, ^4^ Given the similar clinical features of the patients enrolled in these trials, the better performance of CTCs in risk stratification can be extrapolated with caution. On the other hand, the cumulative probability of DM was approximately 19% for the low‐risk group, which is similar to the DM risk in the study groups in both the RAPIDO^3^ and UNICANCER‐PRODIGE 23 trials.^4^ Therefore, our findings indicate that the high‐risk group needs escalation therapy with either a clinical trial with the addition of novel agents or at least FOLFIRINOX‐like regimens. Furthermore, it is important to clarify the mechanism of metastasis for target discovery and drug development. Unfortunately, in this study, the CTCs detected were not further analyzed by single‐cell sequencing. Further studies should focus on this aspect, which could potentially reveal the heterogeneity between primary tumors and metastases.^32^

Notably, the difference in OS was nonsignificant between the two risk groups. Although this does not seem very plausible based on the difference in MFS, it could be attributed to the early detection of metastasis in prospective trials, favorable outcome in treatment for metachronous metastases of CRC and relatively short follow‐up time. In a population‐based study, CRC patients with metachronous liver‐only or lung‐only metastases could achieve 3‐year OS rates of 50.2% and 61.5%, respectively.^33^ In fact, in this cohort, more than 80% of the metastases occurred in the lung or liver only.^34^

Moreover, although the state of MRI‐detected extramural vascular invasion (mrEMVI) has been reported in previous studies to be clearly related to DM,^35^ there is almost a fourfold risk of developing metastases after surgery. This outcome was not observed in the current study, only a trend toward more metastases in mrEMVI‐positive patients, which may be related to the small sample size.

To the best of our knowledge, although the clinical relevance of CTC detection has been broadly reported in CRC, this investigation is one of the very few to uncover the prognostic effect on metastasis of the baseline CTC count for LARC in the neoadjuvant setting. The strengths of our study included the analysis of a subset of data from a randomized trial and the inclusion of a new neoadjuvant treatment modality.

This translational study was limited by the low fraction of patients enrolled among all trial subjects with a limited sample size, the lack of independent validation, and the short follow‐up time for LARC.

In conclusion, for patients with LARC in a neoadjuvant setting, the values of baseline viable CTCs for DM risk stratification and the dynamic change in CTCs for neoadjuvant treatment response prediction were identified in this study. This tool may strengthen the pretreatment risk assessment and postradiotherapy decision making for LARC, which deserves further validation.

## AUTHOR CONTRIBUTIONS

**Wen‐Yang Liu:** Conceptualization (lead); formal analysis (lead); funding acquisition (lead); investigation (lead); methodology (equal); project administration (lead); resources (equal); software (equal); supervision (lead); validation (equal); visualization (equal); writing – original draft (lead); writing – review and editing (equal). **Wen Zhang:** Conceptualization (equal); data curation (equal); formal analysis (equal); investigation (equal); methodology (equal); resources (equal); software (equal); supervision (equal); validation (equal); visualization (equal); writing – original draft (equal); writing – review and editing (equal). **Yuan Tang:** Data curation (equal); investigation (equal); resources (lead); supervision (lead); writing – original draft (supporting); writing – review and editing (equal). **Si‐Lin Chen:** Data curation (equal); writing – original draft (supporting). **Ning Li:** Data curation (equal); writing – original draft (supporting). **Jun‐Qin Lei:** Data curation (equal); writing – original draft (equal). **Jin‐Ming Shi:** Data curation (equal); resources (supporting); writing – original draft (supporting). **Shulian Wang:** Resources (supporting); writing – original draft (supporting); writing – review and editing (supporting). **Ye‐Xiong Li:** Resources (supporting); writing – original draft (supporting); writing – review and editing (supporting). **Kai‐Tai Zhang:** Conceptualization (equal); data curation (equal); formal analysis (equal); investigation (equal); methodology (lead); project administration (equal); resources (equal); supervision (lead); writing – original draft (equal); writing – review and editing (equal). **Jing Jin:** Data curation (equal); formal analysis (equal); funding acquisition (lead); investigation (lead); methodology (equal); project administration (lead); resources (equal); supervision (lead); validation (equal); visualization (equal); writing – original draft (lead); writing – review and editing (lead).

## FUNDING INFORMATION

This study was funded by the Beijing Municipal Science & Technology Commission (no. Z181100001718136), the National Natural Science Foundation of China (no. 82073352), and the Beijing Hope Run Special Fund of Cancer Foundation of China (LC2018A24, LC2022A16).

## CONFLICT OF INTEREST STATEMENT

The authors have no conflict of interest to declare.

## CODE AVAILABILITY STATEMENT

The software used during this study is mentioned in the article.

## ETHICAL APPROVAL

The study was approved by the Ethics Committee of Cancer Hospital, Chinese Academy of Medical Sciences (NO. CH‐GI‐090).

## CONSENT TO PARTICIPATE

Written informed consent was obtained from each patient before enrollment in this study.

## CONSENT FOR PUBLICATION

Written informed consent for publication was not applicable for this study.

## Supporting information


Table S1.

## Data Availability

All data used in this study are available through corresponding author on reasonable request.
