# Supplementary material for: Metastasis risk stratification and response prediction through dynamic viable circulating tumor cell counts for rectal cancer in a neoadjuvant setting
Source: Cancer Med. 2023 Apr 4;12(10):11438–50. doi: 10.1002/cam4.5860 (PMC10242869; doi:10.1002/cam4.5860)
Supplement: Supplementary file 1 — Table S1. [file CAM4-12-11438-s001.docx]

| Variable | Low-risk group  CTCs≤3 (No. and (%))  n=43 | High-risk group  CTCs>3 (No. and (%))  n=40 | P |
| --- | --- | --- | --- |
|  |  |  | .232 |
| Planned Surgery | 36 (83.7) | 25 (62.5) |  |
| Surgery Cancelled Cause |  |  |  |
| CCR | 2 (4.7) | 5 (12.5) |  |
| Metastasis | 2 (4.7) | 7 (17.5) |  |
| Refusal | 4 (4.7) | 2 (2.4) |  |

Table S1 Treatments and short-term results according to CTC risk group after neoadjuvant therapy

CTCs, Circulating tumor cells; cCR, Complete clinical response.
